# Tracing Lipid Metabolism by Alkyne Lipids and Mass Spectrometry: The State of the Art

**DOI:** 10.3389/fmolb.2022.880559

**Published:** 2022-05-20

**Authors:** Lars Kuerschner, Christoph Thiele

**Affiliations:** Life & Medical Science Institute (LIMES), University of Bonn, Bonn, Germany

**Keywords:** click, tracer, analog, probe, lipidomics, fatty acid, metabolism, β-oxidation

## Abstract

Lipid tracing studies are a key method to gain a better understanding of the complex metabolic network lipids are involved in. In recent years, alkyne lipid tracers and mass spectrometry have been developed as powerful tools for such studies. This study aims to review the present standing of the underlying technique, highlight major findings the strategy allowed for, summarize its advantages, and discuss some limitations. In addition, an outlook on future developments is given.

## Introduction

Cellular metabolism generates a vast number of lipid species that form a complex lipid network (Harayama and Riezman, 2018). Most lipids exhibit pronounced hydrophobic properties, favoring their embedding in cellular membranes and cells establish an intriguing spatiotemporal pattern of the various lipids at different membrane loci (van Meer et al., 2008). Their manifold cellular functions and the related pathological malfunctions have rendered lipids a subject of high biomedical relevance (Yang and Han, 2016). Many genetic diseases are based on mutations in enzymes involved in lipid metabolism, remodeling and modification, or in lipid transporters, underlining the importance of lipids in physiology (Harayama and Riezman, 2018). For deciphering the precise role of the various lipids in health and disease, it is mandatory to fully understand the complex metabolic pathways involved. To facilitate research on lipids, they have been systematically categorized and a consensual nomenclature has been established (Fahy et al., 2009; Liebisch et al., 2020).

Studies on lipid metabolism critically depend on suitable research tools. For decades, scientists have been using radiolabeled analogs to trace lipid metabolism along various pathways (Schoenheimer, 1937; Brown and Goldstein, 2009). However, the use of radiotracers in combination with modern lipid analytics, including mass spectrometry (MS), is often impracticable or inconvenient (Batista et al., 2016). Stable isotope tracers have partially substituted here, and several excellent reviews on their applications in lipid research have been written (Liebisch, 2020; Allen et al., 2015; Triebl and Wenk, 2018; Kim et al., 2020).

**FIGURE 1:**
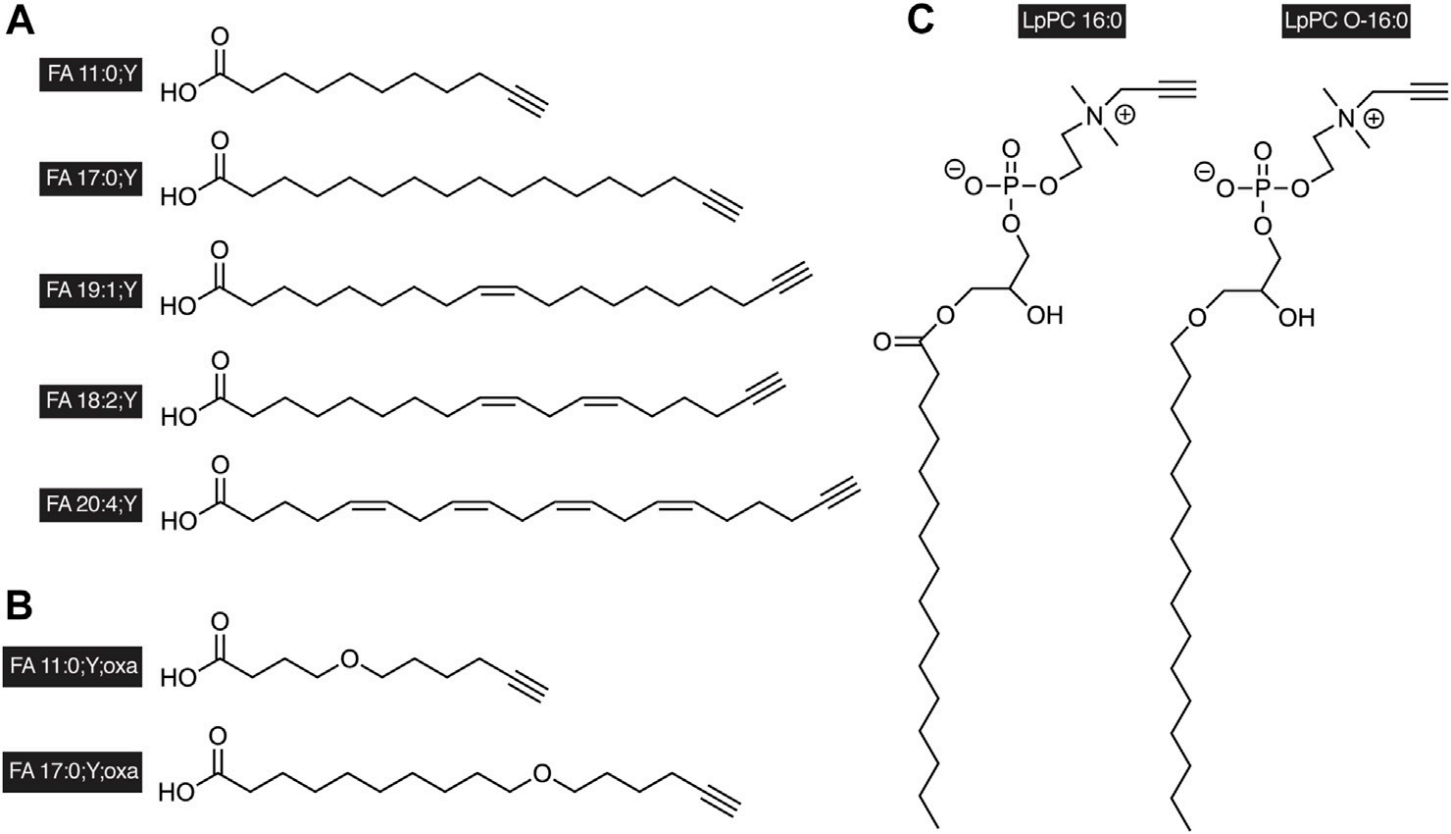
Examples of alkyne-labeled lipids. A terminal triple bond serves as a tag in the side chains of **(A)** alkyne FAs, **(B)** oxaalkyne FAs, or in the head group of **(C)** glycerophosphopropargylcholines. The short nomenclature treats the triple bond as functionalization of the FA as indicated by the suffix “;Y” analogous to a carbon-by-oxygen-replacement denoted by “;oxa.” The propargylcholine head group is described by the lowercase “p” in the name of the lipid class. It is to be noted that alkyne-labeled lipids of various chain length and saturation degrees have been described, both with odd or even carbon counts in the chain.

An alternate replacement for radiolabeled lipids became available in the form of alkyne lipids (Figure 1). These lipid probes contain a single terminal triple bond embedded in their hydrocarbon structure. With the advent of bio-orthogonal chemistry (Bertozzi, 2011) including click chemistry (Kolb et al., 2001), the sensitive and specific detection of compounds containing terminal alkynes has become possible. Copper(I)-catalyzed azide-alkyne cycloaddition, CuAAC, can be used to detect alkynes using azides or vice versa (Rostovtsev et al., 2002; Tornoe et al., 2002). Applying this technique to lipids, including sterols, has yielded numerous examples, where clickable lipids bearing an alkyne or azido tag were successfully used to monitor protein lipidation, protein–lipid interaction, lipid localization and metabolism, and this work has been extensively reviewed (Haberkant and Holthuis, 2014; Kuerschner and Thiele, 2014; Jao et al., 2015; Bumpus and Baskin, 2018; Laguerre and Schultz, 2018; Ancajas et al., 2020; Schoop et al., 2021).

Here, we aim to provide an overview of recent studies using alkyne lipids to trace lipid metabolism by MS. Advantages and limitations of the technique will be discussed, and an outlook on potential applications will be given.

### Alkyne Fatty Acids as Tracers for Lipid Anabolism

Fatty acids (FAs) are a major building block for many lipids. They confer important biophysical properties to the respective lipid molecule and co-determine its biological functions. However, our knowledge of the metabolic dynamics of FAs is limited, in particular on the level of individual lipid species.

MS-based lipidomics is the method of choice for tracing lipids with species resolution (O'Donnell et al., 2020; Züllig and Köfeler, 2021). Discovery lipidomics identifies and quantifies hundreds of individual lipid species from an entire crude extract by either liquid chromatography MS (LCMS) or direct-infusion MS (DIMS) in an untargeted fashion (Han and Gross, 2003; Wenk, 2005; Ståhlman et al., 2009; Harkewicz and Dennis, 2011; Schwudke et al., 2011; Brügger, 2014). In combination with stable isotope tracing, MS lipidomics faces some problems in the unequivocal identification of isotope-labeled lipids in the background of a biological extract. While metabolites of substances with known, limited reactions can be found by targeted analysis, labeled FAs are incorporated into hundreds of products, complicating the analysis (Parks and Hellerstein, 2006). Therefore, available tracing data of isotope-labeled FAs are limited to few labeled species (McCloy et al., 2004; McLaren et al., 2011; Qi et al., 2012).

Alkyne FAs (Figure 1A) carrying a terminal triple bond show a shifted mass value in MS analysis. This mass shift can be used to trace these lipids and their metabolites. However, in DIMS, the depth of their analysis is affected by some intrinsic limitations. The main problem is signal overlap as the mass of the alkyne FA precisely matches that of the corresponding natural FA containing an equal carbon count but two additional double bonds (Milne et al., 2010). This can be overcome by LCMS where alkyne and untagged lipids are chromatographically separated in a procedure that conditionally may include a prior step of alkyne lipid capture and release as dicobalthexacarbonyl complexes (Milne et al., 2010; Beavers et al., 2014; Robichaud et al., 2016). An alternate approach is to use alkyne FA tracers where the triple bond does not replace the terminal single bond but has been added to the hydrocarbon chain, resulting in an odd carbon count and a mass shift of +10 Da (Thiele et al., 2012). Such alkyne FAs have been demonstrated to strikingly mimic their natural pendants in enzymatic processing and cellular metabolism (Thiele et al., 2012; Gaebler et al., 2013; Diehl et al., 2020).

To take full advantage of the alkyne moiety of these tracers for detection, the analytic procedure should wisely benefit from the bio-orthogonal click-reaction with an azide (Kolb et al., 2001; Rostovtsev et al., 2002; Tornoe et al., 2002). For tracing of FA metabolism, this was first achieved by an approach based on the fluorogenic dye 3-azido-7-hydroxycoumarin in combination with TLC separation and fluorescent detection (Thiele et al., 2012). An analytical routine was developed which yielded high linearity and sensitivity and allowed for the detection of sub-picomols of alkyne lipids on a TLC plate. However, the separation by TLC excluded lipid species resolution, prompting the use of this routine in more basic analyses of lipid metabolism.

**FIGURE 2:**
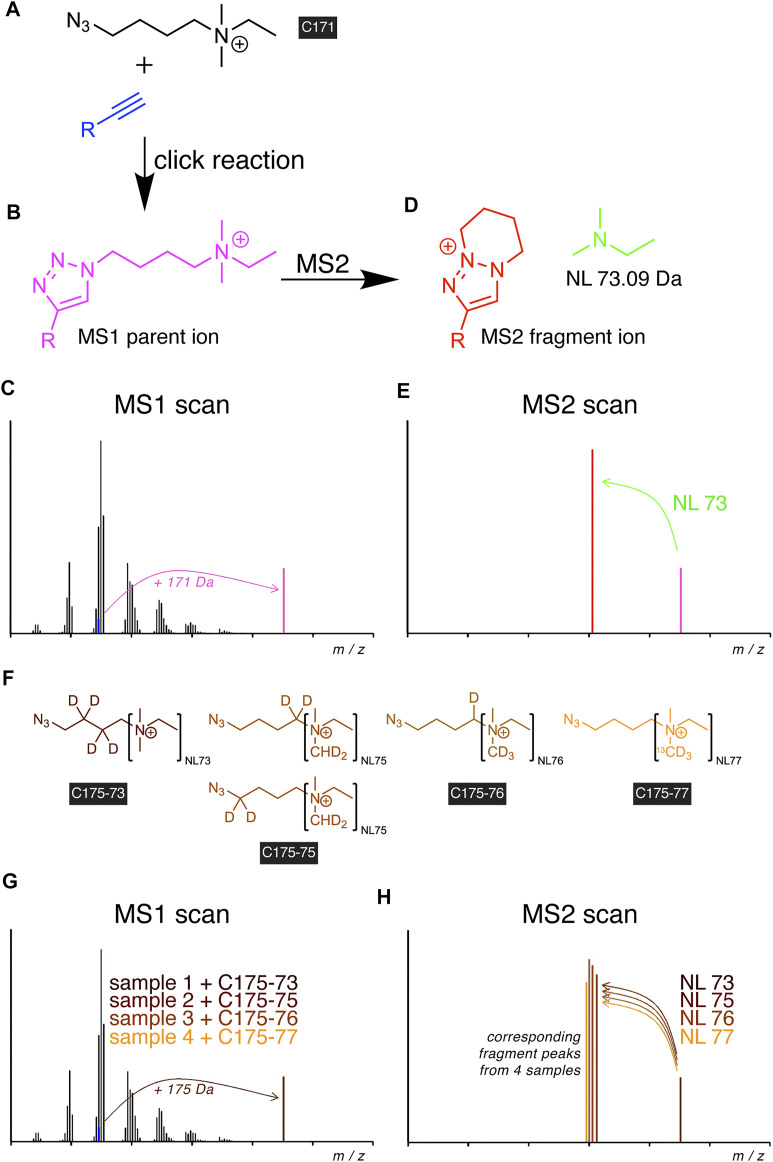
Scheme of alkyne lipid detection by positive-mode DIMS using azide reporters. The C171 reporter **(A)** is click-reacted with an alkyne lipid (blue) to generate a mass-shifted product **(B),** discriminating its MS1 ion peak (**C**, magenta) from unlabeled species of the same lipid class. MS2 fragmentation **(D)** yields a systematic neutral loss (NL, green) and generates a diagnostic fragment ion (**E**, red), enabling the identification of the lipid analyte. For sample multiplexing, the related reporters C175-7x **(F)** are click-reacted to individual samples before sample pooling. The corresponding alkyne lipids from each sample generate a similarly mass-shifted product in MS1 scans **(G)** and are co-analyzed by MS2 but yield unique diagnostic fragment ions **(H)**.

Recently, the idea of also implementing the click reaction into the procedures for detecting alkyne lipids by MS has been put into practice (Thiele et al., 2019). Through the development of a dedicated azide probe termed C171 (Figure 2A), a high-content tracing analysis by MS lipidomics has been empowered. Upon the reaction of the azide with an alkyne lipid in a biological extract, the reaction product carried a permanent positive charge (Figure 2B) that improved its ionization and strongly enhanced the analytic sensitivity to enable the detection of femtomoles of lipids by MS. The additionally conveyed mass shift allowed for direct identification at the MS1 level (Figure 2C). In tandem MS (MS2), the reacted molecule showed a predictable, systematic fragmentation by undergoing neutral loss (NL) of dimethylethylamine (Figures 2D,E). For glycerophospholipids, this NL coincided with the routine NL in the head group (Murphy and Axelsen, 2011).

The uniform fragmentation pattern across all lipid classes in MS2 enabled the implementation of a modified strategy with sample multiplexing capabilities. Similar to the logic in TMT (Thompson et al., 2003) or iTRAQ (Ross et al., 2004) experiments, a set of azide probes, C175-7x (Figure 2F), with additional heavy isotopes distributed over the NL and linker moieties have been developed (Thiele et al., 2019). Upon the click reaction with alkyne lipids of multiple biological extracts, the analogous metabolites from each extract provided identical masses in MS1 (Figure 2G) but different fragments in MS2 (Figure 2H), which allowed the unequivocal assignment of peaks to individual samples in a multiplex mixture. Multiplexing reduced the time needed for analysis by a factor of 4 and removed a major part of stochastic noise originating from liquid handling, spray instabilities, or fluctuations in fragmentation during the analysis.

**FIGURE 3:**
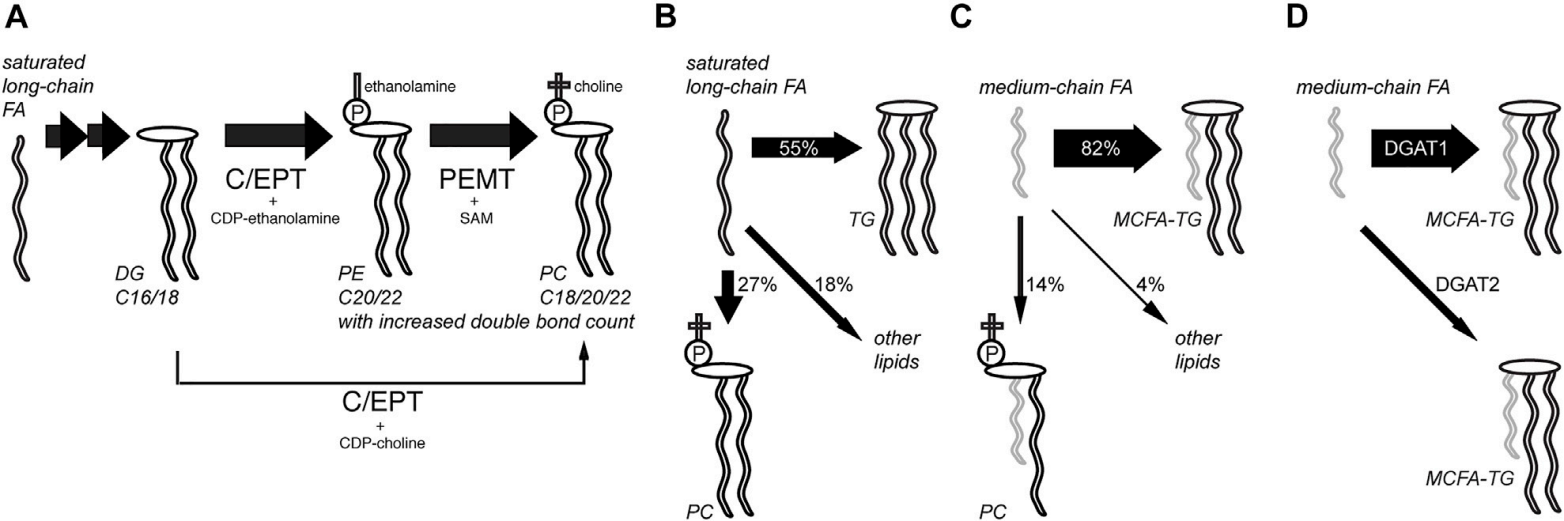
Analysis of hepatic lipid anabolism and remodeling with alkyne FA tracers. **(A)** In pulse-chase experiments using primary mouse hepatocytes and various alkyne FAs, the flow of labels through various lipid classes and lipid remodeling was studied. Hepatocytes produced a major fraction of their abundant polyunsaturated PC species from PE by phosphatidylethanolamine N-methyltransferase (PEMT) using S-andenosyl methionine (SAM), ref. (Thiele et al., 2019). The PE precursor is produced from DG by choline/ethanolamine phosphotransferase (C/EPT) using CDP-ethanolamine as a co-substrate. The more direct pathway from DG to PC with C/EPT using CDP-choline is less prominent in the liver. Following a saturated long-chain FA, this PC synthesis *via* PE is accompanied by lipid remodeling, yielding an increased average side-chain length and double-bond counts over time. The TG and PI pools were also analyzed. **(B,C)** Compared to saturated long-chain FAs that were assimilated quite broadly into various lipid classes after 6 h **(B)**, medium-chain FAs labeled primarily the TG pool **(C)**, ref. (Kuerschner et al., 2022). **(D)** Major part of medium-chain fatty acid TG (MCFA-TG) was synthesized by diacylglycerol acyltransferase 1 (DGAT1) whose activity can only be partially substituted by DGAT2, ref. (Wunderling et al., 2021).

Using this procedure, a set of experiments was performed investigating lipid metabolism in hepatocytes in unprecedented detail (Figure 3, refs. Thiele et al., 2019; Wunderling et al., 2021; Kuerschner et al., 2022). Lipidomics data on the anabolism of palmitate and linoleate were collected using the alkyne tracers FA 17:0;Y or FA 18:2;Y. Also, FA 11:0;Y, an analog of saturated capric acid, was used to investigate differences in the anabolism of mid-chain FAs. The supreme detection sensitivity enabled pulse-chase experiments with very short pulse times (2 or 5 min) that, nonetheless, allowed for the monitoring of hundreds of lipid species during the subsequent chase experiments. Metaphorically, the short-term waves that yielded longer-term ripples in a sea of homeostatic adaptations within the complex lipid network were observed and followed. In turn, this allowed for an evaluation of the contributions of various enzymatic activities.

These experiments showed rapid incorporation of the long-chain FA tracers into early intermediates (PA and DG), and species carrying two copies of the tracers were frequently detected (Thiele et al., 2019). Then, the rapid metabolism of labeled DG to produce TG and PC (the latter *via* PE) occurred, while the labeled PI content increased only slowly during the chase. These later steps in lipid biosynthesis and remodeling varied for different tracers and certain patterns, which depended on the FA length or degree of saturation. Lipids containing the saturated long-chain FA 17:0;Y underwent a steady change toward pairing with longer and more unsaturated FAs. In contrast, most lipids comprising the double-unsaturated long-chain tracer FA 18:2;Y became, over time, more frequently associated with FAs of decreased length and double bond count.

The combination of a saturated and an (poly)unsaturated long-chain FA within a lipid molecule is a common theme (van Meer et al., 2008; Harayama and Riezman, 2018). Saturated medium-chain FAs (MCFAs) with only 8–12 carbons are less abundant in mammals but have become increasingly important as a constituent of certain plant oils in our modern diet (Marten et al., 2006). In the liver, one fraction of the MCFA pool is used for the synthesis of MCFA-containing triacylglycerol (MCFA-TG) and another is used for oxidative energy production or ketogenesis. Using alkyne FA tracers, a recent study investigated which enzymes catalyze the synthesis of MCFA-TG and how an inhibited synthesis or a blocked FA oxidation alters MCFA metabolism in the liver (Wunderling et al., 2021). It was demonstrated that diacylglycerol acyltransferase 1 (DGAT1), and not DGAT2, is the major enzyme for hepatic MCFA-TG synthesis. Specific inhibition of FA oxidation shifted the metabolic flux and led to a compensatory increase in MCFA-TG synthesis.

These studies on primary hepatocytes combined alkyne tracers with pulse-chase experiments and delivered data on FA metabolism at an unprecedented time resolution and sensitivity, reaching 0.2 pmol at the lipid species level (Thiele et al., 2019; Wunderling et al., 2021). By some adaptations of experimental parameters such as a reduction in the sample volume, the limit of quantification was further reduced, down to 0.2 fmol labeled lipid in 20 μL sample volume (10 pM). Hence, the first metabolic tracing experiment with absolute quantification in single cells became possible (Thiele et al., 2019). This single-cell analysis revealed that individual hepatocytes precisely control and quite uniformly maintain their patterns of lipid length and saturation. The apparent robustness in lipid homeostasis resulted in very similar single-cell lipid profiles.

## Alkyne Fatty Acids as Tracers for Lipid Catabolism

FAs contain about one-third of the calories in normal nutrition. This energy is released by cells during FA oxidation. Catabolic β-oxidation is the major degradation pathway for FAs in mammals and co-occurs in cells at the mitochondria and peroxisomes (Van Veldhoven and Mannaerts, 1999; Wanders and Waterham, 2006; Hunt et al., 2012; Houten et al., 2016; Schönfeld and Wojtczak, 2016; Adeva-Andany et al., 2019). The pathway is complex and involves FA activation to an acyl-CoA and organellar uptake, which, in case of mitochondria, includes acyl-CoA conversion to the corresponding acyl-CAR and regeneration of the former inside the organelle. These preparatory steps are finally followed by the actual β-oxidation sequence of enzymatic reactions. In the case of the long-chain FA palmitate, this comprises seven cycles of four enzymatic steps to release eight acetyl-CoA from the initial FA.

**FIGURE 4:**
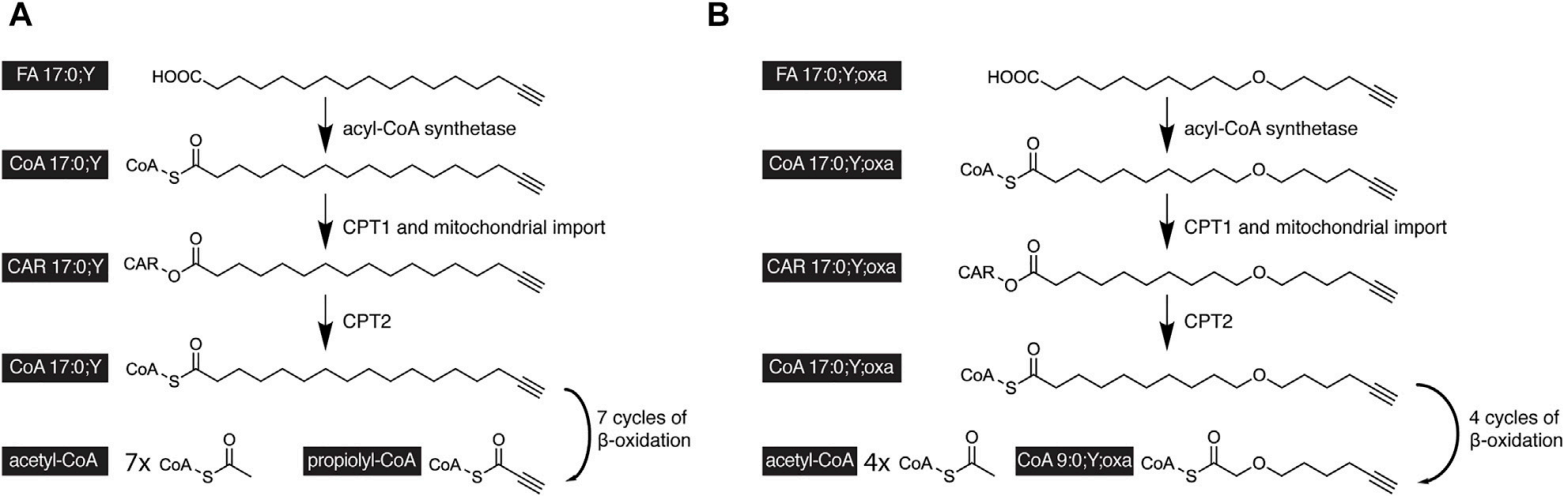
Scheme of the catabolic degradation of alkyne-labeled tracers of palmitate. **(A)** In cells, the alkyne analog FA17:0;Y and **(B)** oxaalkyne analog FA 17:0;Y;oxa are activated by an acyl-CoA synthetase before carnitin palmitoyltranferase 1 (CPT1) forms the corresponding carnitine esters, that are subject to mitochondrial uptake. Intra-mitochondrial CPT2 regenerates the acyl-CoAs, which undergo cyclic β-oxidation. After seven β-oxidation cycles, the FA 17:0;Y yields seven molecules of acetyl-CoA and the unstable propiolyl-CoA **(A)**. After four β-oxidation cycles, the FA 17:0;Y;oxa yields the end product FA 9:0;Y;oxa as CoA thioester containing a stable alkyne label **(B)**.

**FIGURE 5:**
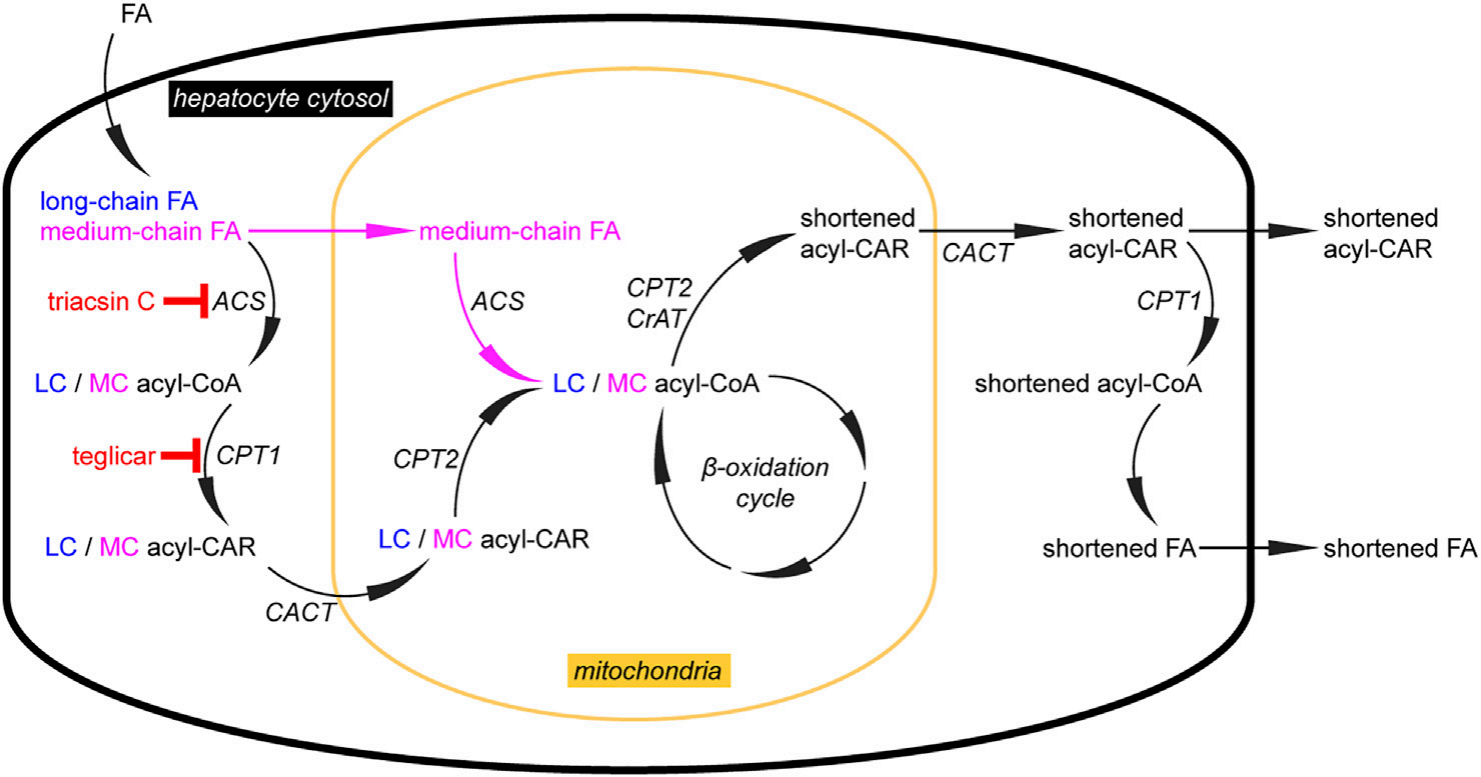
Analysis of hepatic lipid catabolism with alkyne FA tracers. Using alkyne long-chain (LC) and medium-chain (MC) FAs, their different handling in the catabolic pathway of primary mouse hepatocytes was studied. Upon uptake, both FAs follow the pathway (left) with FA activation to an acyl-CoA, conversion to the corresponding acyl-CAR, transport into mitochondria, and regeneration of the acyl-CoA inside the mitochondria. These steps are governed by acyl-CoA synthetases (ACS), carnitine palmitoyltransferase 1 (CPT1), carnitine-acylcarnitine-translocase (CACT), and CPT2, respectively. When triacsin C or teglicar inhibit the cytosolic steps, only MCFAs can enter the mitochondria in the unesterified form, before activation by a mitochondrial ACS (that appears unaffected by triacsin C). In the mitochondrial matrix, these preparatory steps are followed by the actual β-oxidation cycle. This sequence of enzymatic reactions does not proceed completely successively for a single FA molecule but rather generates a pool of intermediates that is sufficiently long-lived to be accessible for CPT2 or carnitine acetyltransferase (CrAT) to generate shortened acyl-CARs that are released from the mitochondria to the cytosol (right). There, they undergo transesterification, forming acyl-CoA for various metabolic pathways, including secretion from cells. Of note, facilitated by an unidentified transporter, hepatocytes also secrete the shortened acyl-CARs. These metabolites may also fulfill a signaling function as they carry information about the metabolic status of this major β-oxidizing tissue, ref. (Kuerschner et al., 2022).

Upon complete β-oxidative processing, an alkyne FA yields a short-chain terminally unsaturated CoA thioester. Accordingly, the metabolism of the alkyne analog of palmitate, FA 17:0;Y, yields propiolyl-CoA (Figure 4A). This end product has been found unstable (Patel and Walt, 1988) and thus escaped accurate detection and quantification. To overcome this potential drawback, oxaalkyne FAs (Figure 1B) have recently been introduced as dedicated tracers to study β-oxidation (Kuerschner et al., 2022). Here, an oxygen atom replaces a carbon atom within the hydrocarbon chain, and during catabolic shortening of the chain, the substituting oxygen eventually arrests further processing (Figure 4B). This led to the accumulation of a pathway end product that carried a stable alkyne moiety and was easily detectable. In addition, a distinct set of intermediates could be followed over time. That way and focusing on hepatic FA catabolism, the study confirmed differences in metabolic handling of long- and medium-chain FAs (Figure 5). Unlike the longer ones, medium-chain FAs that were activated inside or outside of the mitochondria by different acyl-CoA synthetases entered the mitochondria as free FAs or carnitine esters. Upon mitochondrial β-oxidation, shortened acyl-carnitine metabolites were produced and released from the mitochondria. Hepatocytes ultimately also secreted these shortened acyl chains. When mitochondrial β-oxidation was hindered, peroxisomal β-oxidation acted as a salvage pathway and maintained the levels of shortened FA secretion.

## Head Group Labeling

Membrane lipids regularly contain head groups that feature hydrophilic or charged substructures. While conferring identity parameters to the lipids and co-guiding their differentiation into lipid classes, the various head groups also profoundly contribute to the many physiological roles of lipids (van Meer et al., 2008; Fahy et al., 2009; Harayama and Riezman, 2018). For head group tagging that uses the click reaction, different strategies exist. First, an azide moiety at the tracer head group may be used for subsequent detection by alkyne reporters. Such swapping of functionalities between the click partners is readily possible, and as a component of the head group, the partially charged azide moiety often is well-tolerated by the biological apparatus. Applications of various azide-tagged head groups in studies on lipid metabolism and other topics have recently been reviewed (Bumpus and Baskin, 2018; Ancajas et al., 2020).

Second, also, the alkyne moiety can be accommodated in the head group. This approach has been used for the phosphocholine head group to study the metabolism of the phosphocholine-containing lipids, PC, PC O, and SM. The original strategy used propargylcholine, a choline analog bearing a terminal alkyne, for metabolic labeling of the phosphocholine-containing lipids (Jao et al., 2009). The water-soluble alcohol was administered to the biological specimen and entered lipid metabolism *via* the Kennedy pathway or PLD remodeling (Bumpus et al., 2018). Alternatively, a labeling strategy using synthetic lyso-phosphatidylpropargylcholine LpPC or its ether pendant LpPC O was used to specifically target the PC or PC O pool, respectively (Yaghmour et al., 2021).

**FIGURE 6:**
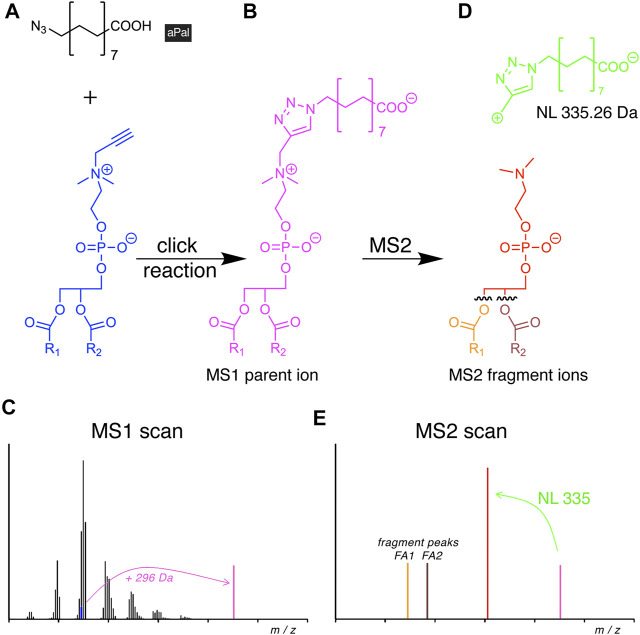
Scheme of alkyne lipid detection by negative-mode DIMS using azidopalmitate (aPal). The aPal reporter **(A)** is click-reacted with a phosphatidylpropargylcholine (blue) to generate a mass-shifted product **(B),** discriminating its MS1 ion peak (**C**, magenta) from unlabeled phosphatidylcholine species. MS2 fragmentation **(D)** yields a stereotypical neutral loss (NL, green) and generates a diagnostic fragment ion (**E**, red) that upon further fragmentation not only enables the identification of the lipid analyte, but also of the two FA chains.

The resulting propargylcholine metabolites were traced along metabolic pathways by MS, benefiting from a specific precursor ion in the positive ion mode (Jao et al., 2009; Bumpus et al., 2018; Paper et al., 2018). However, this approach only delivered their sum FA composition. This limitation has recently been overcome with the introduction of a novel azide reporter, azidopalmitate (Figure 6A), for propargylcholine-containing phospholipids (Yaghmour et al., 2021).

Upon the click reaction, the tagged lipids carried a permanent negative charge (Figure 6B) that improved their ionization and enhanced the method’s sensitivity to enable the detection of picomoles of lipids. The additionally conveyed mass shift allowed for direct identification at the MS1 level (Figure 6C). In MS2, the reacted molecule showed stereotypical fragmentation by undergoing a characteristic NL (Figure 6D). As the analysis was performed in the negative mode, it also disclosed the identity of the FA side chains (Figure 6E). The new method was used to investigate the cellular PC and PC O content. The study revealed differences in pool size, apparent metabolic stability, and side-chain composition of both lipid classes by providing a quantitative picture of their metabolism and homeostasis (Yaghmour et al., 2021).

Propargylcholine also served as a head group label for tracing SM metabolism involving sphingomylinase and sphingomyelin synthase (Jao et al., 2009; Sandbhor et al., 2009; Yaghmour et al., 2021). In addition, for PA an alkyne head group label, 6-hexynol has been described (Bumpus and Baskin, 2016). Here, the tagged PA molecule was generated from glycerophospholipids by phospholipases D using 6-hexynol, rather than water as the co-substrate.

## Standards for Quantification

Absolute quantification is highly desired in lipidomics. This is achieved by internal standardization using synthetic lipids, here alkyne lipids. There are several possibilities for using uniquely labeled alkyne lipids as internal standards. First, a standard of dissimilar carbon count can be used. For instance, if the experiment uses a tracer with an even number of carbon atoms in the side chain, a set of internal standards featuring an odd carbon count can often be used or vice versa. This strategy follows the notion that most metabolites of the tracer used will have the same side chain carbon count as the tracer or one that varies by multiples of 2. While this is by and large correct for standard mammalian metabolism, some mammalian cell types (e.g., adipocytes) and most bacteria contain significant amounts of odd-numbered FAs that interfere with this standardization strategy.

Alternatively, isotope-labeled alkyne lipids can be used as internal standards. For this, isotopes with D or ^13^C have been proven to be most valuable. It is worth mentioning that even when high-resolution MS equipment is available, the deuterated standards used should contain at least three D atoms to allow for unequivocal identification of the lipid with reliable discrimination from ^13^C isotope peaks by high-resolution MS.

In any case, for best data quality, all internal standards should be added to the sample at the earliest possible step during sample preparation, that is, as part of the extraction mix and at a quantity that fairly matches the expected concentration of the analyte. Naturally, the internal standards should also contain an alkyne moiety to monitor the performance of the click-reaction and all other steps of sample processing and analysis.

## Conclusion

Investigations on lipid metabolism greatly benefit from the use of lipid analogs. Using any analog in an experimental study, one has to be aware of the probe limitations. Lipids are relatively small molecules, often with complex physicochemical and biological properties that are not easily matched to the full extent by their analogs. Radio- and isotope-labeled lipids represent structurally optimal analogs, allowing for very sensitive detection and have proven invaluable for studies on lipid metabolism. Regarding alkyne analogs, it has to be noted that here a tag, albeit a small one, that consists of a single triple bond is embedded in the lipid structure. Unlike other, for example fluorescent tags, the diminutive alkyne group usually shows little to no impact on important metabolic properties (Thiele et al., 2012; Alecu et al., 2017). However, in the case of FA 20:4;Y, a surrogate of arachidonate, some deviations in the metabolism were demonstrated that affected ω-oxidation and eicosanoid synthesis in particular (Beavers et al., 2014; Robichaud et al., 2016). In general, ω-oxidation should better not be studied using alkyne FAs as their terminal triple bond likely interferes with this pathway’s enzyme activities (Ortiz de Montellano and Reich, 1984).

By now, many alkyne analogs of different classes of lipids (fatty acyls, glycerolipids, glycerophospholipids, sphingolipids, sterols, and prenols) have been described and are commercially available. In addition, isotope-labeled lipids have become widely purchasable, however, mostly as deuterated analogs, while much fewer lipids carrying ^13^C or ^15^N can be obtained from commercial sources (Liebisch, 2020). The ^13^C-labeled tracers are often preferred over the deuterated lipids as their label is more stable in a protic solvent and during metabolic desaturation reactions (Triebl and Wenk, 2018). It is worth mentioning that it is possible to biologically incorporate an isotope label very broadly into a wide spectrum of metabolites, including lipids, by the use of isotope-labeled glucose, glycerol, amino acids, or D_2_O. No equivalent for an alkyne labeling strategy is possible. However, despite the cost-effectiveness of such generalized label incorporation, often more pathway-specific tracers are preferred. To satisfy these needs, it is important to point out that a large and diverse selection of alkyne lipids is relatively easily, conveniently, and cost-efficiently producible by a skilled chemist.

A powerful feature of alkyne lipids is their suitability for fluorescence microscopy. Using the same tracer, this allows for parallel investigations on lipid metabolism and distribution. The lipid’s localization can be visualized by fluorescent microscopy using azido-fluorophores or azido-biotin with fluorescent streptavidin (Jao et al., 2009; Hofmann et al., 2014; Gaebler et al., 2016). Azido-biotin in combination with gold probes also allows for a localization of alkyne lipids by electron microscopy, offering the highest spatial resolution (Jao et al., 2009; Iyoshi et al., 2014). These applications have recently been reviewed (Haberkant and Holthuis, 2014; Kuerschner and Thiele, 2014; Bumpus and Baskin, 2018; Laguerre and Schultz, 2018; Ancajas et al., 2020). While RAMAN and MS imaging have significantly advanced in resolution, optical and electron microscopy still excel in spatial resolution by several orders of magnitude (Berry et al., 2011; Wei et al., 2016; Jamieson et al., 2018; Bowman et al., 2019; Porta Siegel et al., 2019; Tuck et al., 2021). With their applicability to all imaging techniques, alkyne lipids appear more suitable than isotope-labeled lipid tracers for most localization studies.

The recent developments of novel azide reporters and detection procedures have unleashed the power of alkyne analogs for tracing lipid metabolism. Altogether, this technology has opened new avenues for high-content lipid analysis by MS lipidomics. It should be stressed that the technology enables lipid-tracing studies in *in vitro* systems and in biological specimens of all levels, including cells, tissues, and whole animals. The depth of information gained will allow for large-scale network modeling of entire pathways, and such studies have been initiated. Surely, future developments of the technique will include multilabeling experiments, where the metabolic fate of several alkyne tracers in a single biological experiment will be followed in parallel. Similarly, correlative studies on alkyne lipid metabolism and localization will also be performed, potentially involving mass spectrometry imaging.
